# Diagnostic and Prognostic Value of Laboratory Risk Indicator for Necrotizing Fasciitis Score

**DOI:** 10.7759/cureus.37775

**Published:** 2023-04-18

**Authors:** Nimil Mary Thomas, Minaxi Sharma, Mukta Sukhadia, Ardra Merin George

**Affiliations:** 1 General Surgery, Rabindranath Tagore (RNT) Medical College, Udaipur, IND; 2 Surgery, Rabindranath Tagore (RNT) Medical College, Udaipur, IND; 3 Preventive Medicine, Government Medical College & Hospital, Kozhikode, IND

**Keywords:** sepsis, mortality, soft tissue infection, lrinec, necrotizing fasciitis

## Abstract

Background: Necrotizing fasciitis (NF) is a lethal soft tissue infection involving skin and subcutaneous tissue with significant morbidity and mortality.

Aim: To validate the diagnostic and prognostic role of the Laboratory Risk Indicator for Necrotizing Fasciitis (LRINEC) scoring system for NF in patients who present with soft tissue infections.

Methods: The study was conducted on 100 patients who presented with soft tissue infections. Based on the histopathological findings, they were divided into NF and non-necrotizing soft tissue infection groups. Patients were clinically assessed. The lab parameters were assessed and the LRINEC score was calculated. Patients were stratified based on score and grouped into low, intermediate, and high risk. For patients who went into sepsis, the death rate and length of hospital stay, including ICU, were noted based on the scoring system.

Results: In our study, the diagnostic role of LRINEC score ≥ 6 had a sensitivity of 85.7% and specificity of 62.7%, and score ≥ 8 had a sensitivity of 67.3% and specificity of 82.3% with a positive predictive value (PPV) of 78.5 and negative predictive value (NPV) of 72.4, of which 8 is a better cut-off as a diagnostic criterion. The area under the curve was found to be 0.835. To predict the prognostic role, a cut-off value was calculated from the receiver operating characteristic curves of both mortality and sepsis patients in relation to the LRINEC score of 9. With the LRINEC score cut-off as 9, with mortality and sepsis as variables, the sensitivity was 50% and 53.3%, specificity was 94.2% and 91.4%, PPV was 78.9% and 72.7%, and NPV was 81.4% and 82%, respectively.

Conclusion: The LRINEC score is quick, safe, reproducible, noninvasive, cost-effective, and easily calculated, and has high sensitivity and specificity to predict early diagnosis, and it could be used for risk stratification and prognosis of necrotizing soft tissue infections.

## Introduction

Necrotizing fasciitis (NF) is a rare, potentially life-threatening infection involving subcutaneous tissue and fascia [1,2]. It can progress to necrosis leading to septic shock and even death. The incidence of NF in adults is approximately four per 100,000 individuals [3-5].

Despite many advances in our understanding of this disease and great improvements in medical care, mortality associated with NF has not changed in the last 30 years and remains globally up to 40%, with around 500 cases per year, and in India, the mortality rate is 75% with amputation rate of 15-30% [6]. The frequent cause of necrotizing soft tissue infection is usually trivial trauma or surgical wound. However, no definitive causes can be found in 20-50% of cases. For patients who have decreased immunity or other comorbid conditions, such as diabetes mellitus, cirrhosis, malignancy, chronic renal failure, obesity, smoking, and alcoholism, corticosteroid therapy is associated with necrotizing soft tissue infection [7-10]. NF is commonly localized in the lower extremities, trunk, and perineum [11-13]. Classic physical examination signs, which have been proposed to differentiate NF from non-necrotizing soft tissue infection, include excruciating pain, blister, crepitus, edema other than erythema, and rapidly spreading hemorrhagic bullae [14].

Hematological changes in NF are similar to septic patients. These changes include leukocytosis, leucopenia, coagulopathy, and thrombocytopenia. Anemia can be dilutional from fluid resuscitation or from hemolysis. Disseminated intravascular coagulation is common in severe sepsis. Raised serum creatinine indicates myositis or myonecrosis, circulating toxins, or ischemia. Hypocalcemia is a sign of fat necrosis and calcium deposit in necrotic tissues. Bacterial infection, inflammation, and necrosis cause raised C-reactive protein (CRP). As in severe sepsis, abnormal renal function, hypoalbuminemia, hyponatremia, abnormal liver function, metabolic acidosis, and high serum lactate concentrations may occur [15].

Our study is focused on the diagnostic and prognostic value of the Laboratory Risk Indicator for Necrotizing Fasciitis (LRINEC) score so that necrotizing and non-necrotizing soft tissue infections can be differentiated, and early diagnosis followed by surgical interventions can be done. Additionally, the prognostic role of the LRINEC score is also evaluated based on the outcome of the patient in terms of length of the hospital stay, including ICU, sepsis, and mortality.

## Materials and methods

Data collection

This cross-sectional and observational study was conducted at Maharana Bhupal Government Hospital, Udaipur, India in the Department of General Surgery on 100 patients for one year after the study protocol was approved by the Institutional Review Board and the Ethical Clearance Committee (RNT Medical College and Controller and Attached Hospitals, Udaipur; approval number: RNT/Stat/IEC/2021/843). The clinical data for this study were obtained from patients who had given consent, were symptomatic, and were admitted to the Department of General Surgery with soft tissue infections. Patients were selected by sampling technique based on inclusion and exclusion criteria.

Inclusion criteria

Inclusion criteria included patients presenting with symptoms suggestive of soft tissue infections during the study period.

Exclusion criteria

The exclusion criteria were as follows: patients under 15 years; patients who have undergone surgical debridement for the present episode of soft tissue infection; patients with boils or furuncles with no evidence of cellulitis; patients who were known cases of inflammatory bowel disease, rheumatoid arthritis, and lupus (CRP is raised in these patients).

Each patient record was analyzed based on demographic information, predisposing factors, comorbidities, anatomical site of infection, and microbiological findings. Diagnostic confirmation and patient stratification into group 1 (necrotizing fasciitis) and group 2 (non-necrotizing soft tissue infections) were based on operative findings such as the greyish color of the debrided necrotizing fascia, easy detachment of normally adherent fascial planes, lack of bleeding on the cut surface of the involved fascia, presence of dish wash pus odor, and confirmation based on histopathology. Laboratory findings were analyzed and LRINEC scores were calculated at the time of admission, which is based on six variables, namely, hemoglobin (Hb), WBC count, serum sodium (Na), serum creatinine, serum CRP, and random blood sugar. The patients were grouped based on the score: low ≤ 5, intermediate = 6-7, and high risk ≥ 8. Re-evaluation of patients was done at an interval of six hours for two days until no further necrosis was visible, and all infective foci were removed. For sepsis, the quick Sequential Organ Failure Assessment (q-SOFA) score was calculated based on the Glasgow Coma Scale, respiratory rate, and blood pressure. The prognosis was contemplated on the basis of the length of hospital stay, including ICU stay, sepsis, and mortality. Standard treatment after admission included surgical debridement and fasciotomy, as well as broad-spectrum antibiotics based on microbiological findings.

Statistical analysis

Full statistical analysis was performed using SPSS version 18 software (SPSS Inc., Chicago, IL). Variables were assessed by mean and standard deviation after checking for normality in the preliminary analysis. For the variables that were not normally distributed, the median and range were reported. Analysis was conducted using the Student's t-test or ANOVA test for continuous variables and Pearson chi-square test for categorical variables. The receiver operating characteristic (ROC) curve was plotted for the four variables found to be significant and used to determine the cutoff for LRINEC score to determine the diagnostic accuracy and also ROC in terms of sepsis and mortality to predict the prognostic role.

## Results

During our period of study (sample size = 100), 49 cases were of NF and 51 of them were of cellulitis (Table 1).

**Table 1:** HPE vs. LRINEC score HPE: histopathological examination; LRINEC: Laboratory Risk Indicator for Necrotizing Fasciitis.

Score group	Necrotizing fasciitis	Cellulitis	Chi-square	P-value
High risk ≥ 8	33	9	29.76	0.00
Moderate risk = 6-7	9	10		
Low risk ≤ 5	7	32		

The most common age group of the study population was in the range of 40-79 years, more predominantly 60-79 years. In the overall study population, males were predominant. Statistically, the gender factor is significant among the NF group. Farmers were predominantly affected in our study population. In our study sample, the most common risk factor leading to both necrotizing soft tissue infection and non-necrotizing soft tissue infection was trauma. Lower limbs were most commonly affected by the disease process in both NF and cellulitis followed by the scrotum in the NF population. *Escherichia coli* was the most common bacteria isolated in our study population followed by *Klebsiella* in the NF group. The median LRINEC score in NF patients was found to be 6.

Patients were categorized according to the LRINEC score into high, moderate, and low-risk categories. It was observed that the higher the score, the greater the risk of NF, and the correlation was found to be statistically significant. It was also observed that in the high-risk group (i.e., score ≥ 8), values of Hb, CRP, Na, and total WBC count were statistically significant in the NF group (Table 2). Most of the patients were managed by debridement, among which NF group patients were managed by both debridement and fasciotomy.

**Table 2:** Correlation between LRINEC score and variables LRINEC: Laboratory Risk Indicator for Necrotizing Fasciitis.

Variable	≥8 (n = 42)	6-7 (n = 19)	<5 (n = 39)	P-value
Hemoglobin	9.8 ± 2	11.9 ± 3.4	11.8 ± 2.1	0.001
C-reactive protein	37.2 ± 24.9	19.4 ± 9.3	13.4 ± 9.5	0.000
Sodium	131 ± 6.4	133 ± 4.7	135 ± 9.5	0.013
Total white blood cell count	23,281 ± 13,486	15,683 ± 10,617	11,317 ± 4,783	0.000
Random blood sugar	127 ± 94	109 ± 47	130 ± 93	0.604
Creatinine	1.9 ± 1.2	1.4 ± 0.8	1.2 ± 0.89	0.15

LRINEC score as a diagnostic modality

LRINEC score as a diagnostic modality was found to be statistically significant (Table 3).

**Table 3:** Comparison of LRINEC cut-off scores as a diagnostic modality LRINEC: Laboratory Risk Indicator for Necrotizing Fasciitis.

LRINEC cut-off score	≥6	≥8
Sensitivity	85.7%	67.3%
Specificity	62.7%	82.3%
Positive predictive value	68.8%	78.5%
Negative predictive value	82%	72.4%

**Figure 1:**
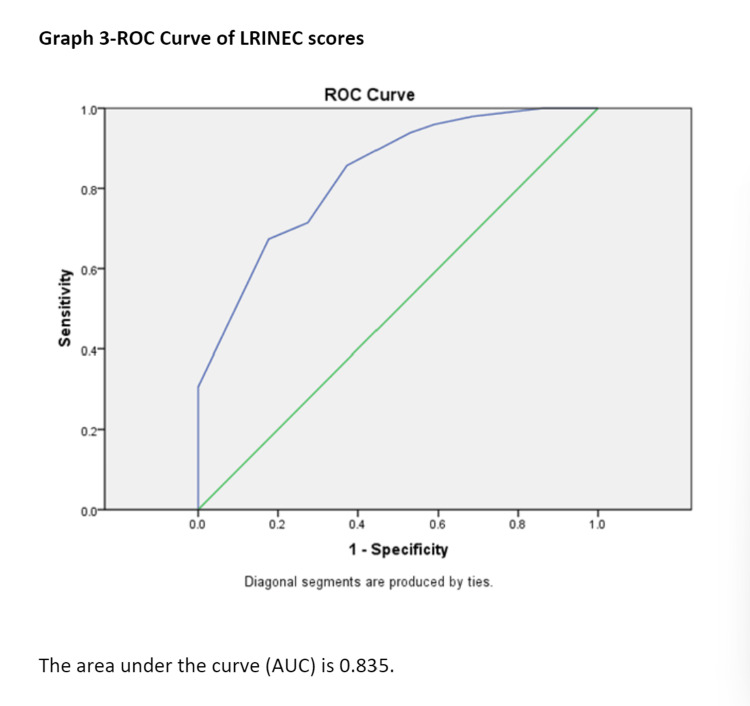
ROC curve of LRINEC score ROC: receiver operating characteristic; LRINEC: Laboratory Risk Indicator for Necrotizing Fasciitis.

The higher the score, the more the chances for NF. According to the ROC curve, the best cutoff for the LRINEC score is 8, as it is a discrete variable with a specificity of 82.4% and sensitivity of 67.3%. The area under the curve was 0.835. Thus, it can be used as a diagnostic modality when the LRINEC score is ≥8 (Figure 1).

LRINEC score as a prognostic indicator

**Figure 2:**
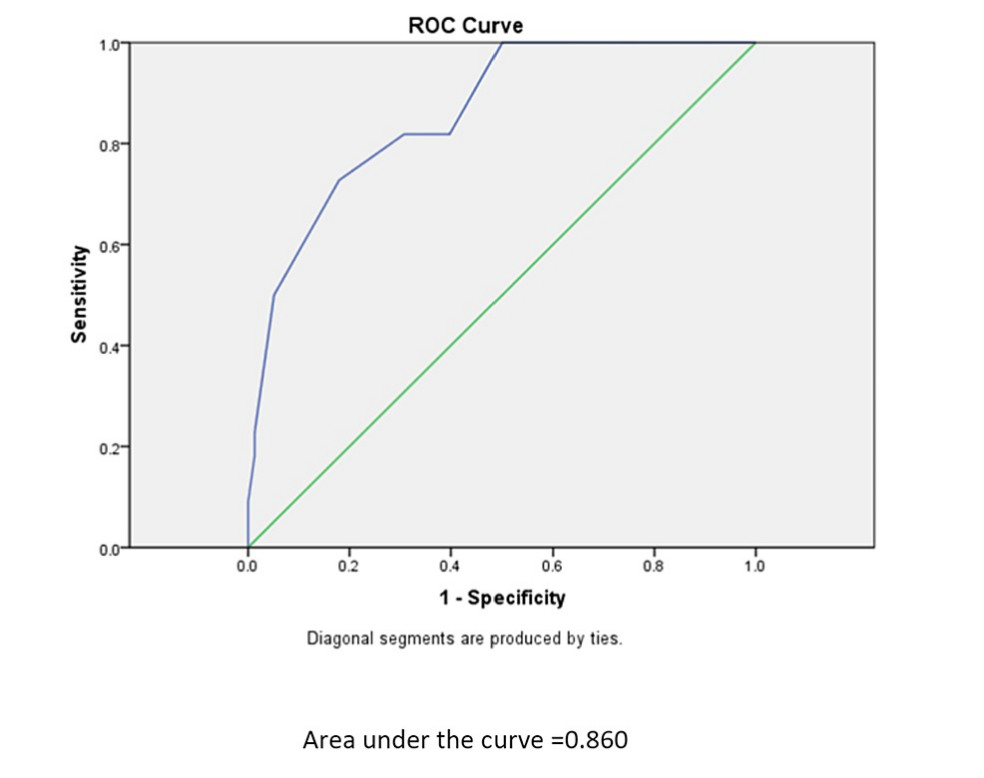
ROC curve of LRINEC score in terms of sepsis ROC: receiver operating characteristic; LRINEC: Laboratory Risk Indicator for Necrotizing Fasciitis.

Sepsis was seen in the majority of the patients in the high-risk group of NF with a score of ≥8 and it was found to be statistically significant, hence ROC curve was plotted. The area under the curve (AUC) was 0.860 (Figure 2).

The cut-off LRINEC score was 9 with a sensitivity of 72.7% and a specificity of 82.1%. When the LRINEC cut-off was taken as 6, the specificity was 50% with a negative predictive value (NPV) of 100%, but with a cutoff of ≥ 9, the specificity was 91.4% with an NPV of 82%. With sepsis, mortality and the number of hospital days were also evaluated. The correlation between death and NF was found to be statistically significant, and AUC was 0.865 with a cut-off ≥ 9, sensitivity of 78.9, and specificity of 81.5% (Table 4).

**Table 4:** Comparison of LRINEC score cut-off of 9 (sepsis and mortality) LRINEC: Laboratory Risk Indicator for Necrotizing Fasciitis.

LRINEC cut-off	≥9, Mortality	≥9, Sepsis
Sensitivity	50	53.3
Specificity	94.2	91.4
Positive predictive value	78.9	72.7
Negative predictive value	81.4	82

The majority of deaths of patients in the NF group were in the first week of admission. On analyzing the above table, patients who were admitted to the hospital after a week of presentation of symptoms had a mortality rate and sepsis rate of 100%. The outcome of the patient and score was found to be statistically significant. The lower-score patients were discharged with good healing, and a greater number of deaths were seen in patients with scores ≥ 8. On analysis, it was found that the higher the score (>6), the more the length of hospital stay.

## Discussion

In patients with NF as a preliminary diagnosis, risk stratification and prompt appropriate interventions in terms of broad-spectrum antibiotics and surgical debridement along with fasciotomy are pivotal for better outcomes. Despite improvements in the diagnosis and management, this rapidly progressive infection remains associated with a high mortality rate of 10.9-76%. The silver lining is that mortality is directly proportional to the time of intervention [13,15]. Since MRI and frozen section biopsy cannot be done often to diagnose and monitor the disease course, the LRINEC scoring system can be utilized, which includes both clinical and laboratory variables for the diagnosis, to determine the disease progression and the outcome of the patient, and if not done, the risk of missing early cases of NF increases, which can totally affect the outcome. There are many literature studies regarding the diagnostic role of the LRINEC score but there are only very few studies that have reported the prognostic role of the LRINEC score. Our study population was 100, out of which 49 cases were of NF and 51 were of cellulitis. In the present study, males were predominantly affected, with a mean age of presentation of 54 years, which was consistent with other studies [16-22]. The other significant finding in our study was the history of trauma leading to NF in contrast to other studies where diabetes mellitus was the most common comorbidity and risk factor according to other studies [8,23] in patients of NF, which may account for outdoor activities of middle-aged males of rural background who are more exposed to trauma and other risk factors. On analyzing the demography, it is clearly seen that the majority of our sample group members were farmers by occupation who are exposed to frequent minor traumas in their day-to-day life, which can be a predisposing factor for NF. An inciting event cannot be detected in 45% of cases of NF, and these are called primary or idiopathic NF [24]. If NF appears after a known etiology, it is called secondary NF and usually occurs due to skin trauma such as minor trauma, insect or snake bite, improper injection, burn, perianal abscess, and skin ulcer [25]. So in our study population, the majority of cases were of secondary NF. The majority of NF patients in our study involve the lower limb extremities followed by Fournier's gangrene, with *E. coli* as the most commonly isolated microorganism in 44% of cases. These results were consistent with other studies [17,18,26].

In 2004, Wong et al. [27] described a diagnostic scoring system named LRINEC “Laboratory Risk Indicator for Necrotizing Fasciitis" with a sensitivity of 90%, specificity of 95%, and an NPV of 93%. LRINEC score, although subject to controversy in previous studies [27], still excludes significant diseases with a low-risk score. To date, the highest sensitivity for the LRINEC was found in the original study by Wong et al. (2004) at 89.9% [27]. In our study, LRINEC score ≥ 6 had a sensitivity of 85.7% and specificity of 62.7% and LRINEC ≥ 8 had a sensitivity of 67.3% and specificity of 82.3% with a positive predictive value (PPV) of 78.5 and NPV of 72.4, of which 8 is a better cutoff as a diagnostic criterion. The AUC of the LRINEC ROC curve was found to be 0.835, which predicts that the LRINEC score is a good diagnostic tool for NF patients.

When individual variables were plotted in the ROC curve, a statistically significant correlation was established with WBC, Hb, Na, and creatinine, thus it can be concluded that patients with anemia (Hb < 10 g/dl), raised total white blood cell count (total WBC > 16,000 µL), raised CRP (>15 mg/dl), and hyponatremia (Na < 131 meq/L) harbor significant risk for progression to NF. It was found that among the 49 patients, 33 patients had NF with a score of more than 8, which is the high-risk group and was proven statistically significant.

The average time from onset to presentation to our hospital was five days, which was a concern. In our study, it was found that there was a statistically significant correlation between the onset to presentation with sepsis and mortality. More delays in presentation to the hospital increased the morbidity and mortality of the patients. This may be due to the remotely placed rural population with decreased medical literacy rate and limited health infrastructure with limited resources at primary health centers. In our healthcare sector, most of the patients with cellulitis are admitted to the medical team, which also delays early intervention. This was in accordance with previous studies [15,28]. Along with the delayed presentation, the factors that contribute to the high rate of sepsis and mortality rates are increasing age, male gender, trauma, low hemoglobin, high serum CRP and total WBC count, hyponatremia, gangrene, and fascial necrosis in histopathological examination.

To evaluate the prognostic role of LRINEC score in NF, sepsis and mortality were taken into consideration. Sepsis was defined on the basis of the q-SOFA scoring system where a score ≥ 2 was suggestive of sepsis [29]. The rate of complications (sepsis, transfer to intensive care, and mortality) in NF patients with LRINEC ≥ 6 was higher when compared to the patients with a score < 6. Our study reports significantly higher mortality and sepsis rates in patients with LRINEC scores ≥ 6. In addition, the duration of stay in the hospital, including Ithe CU, was significantly longer among patients with higher scores. These observations were consistent with previous studies [23,24,30] and were helpful in predicting the prognosis of the patient and may act as a guiding factor for appropriate and timely intervention. To predict lethality, a cut-off value was calculated from the ROC curves of both mortality and sepsis patients in relation to the LRINEC score of 9 as it is a discrete variable. It was observed that the LRINEC score ≥ 9 has a sensitivity of 50%, specificity of 94.2%, PPV of 78.9%, and NPV of 81.4%. It was also observed that albumin and lactate dehydrogenase were significantly affecting the prognosis of patients in NF, thus these variables can also be added to the score in terms of determining the prognosis of NF patients.

Early presentation, early referral, well-equipped primary healthcare facilities, awareness in rural population, and education regarding the scoring system to healthcare workers would be helpful in the stratification of patients and thus prompt intervention. With the above-mentioned significant observation, it seems plausible to apply this scoring system both at the primary and tertiary centers to encompass the relevant population. This would help us to stratify and manage the patients along the lines of early referral, appropriate sequential and synergistic treatment options, early predictors of the course of progression of NF, and apropos management, thus significantly reducing both morbidity and mortality.

Limitations

Our study group sample size was very small, hence the statistical significance of all the variables could not be established. We did not differentiate the extent of body involvement, which may have influenced the laboratory parameters and the score. Serial LRINEC scores were not calculated in patients, as it can also establish a new correlation in the outcome of the patients.

## Conclusions

The LRINEC score is quick, safe, reproducible, noninvasive, cost-effective, and easily calculated, and has high sensitivity and specificity to predict the early diagnosis of necrotizing soft tissue infection. In addition to its early diagnostic role, it could be used for risk stratification and prognosis of necrotizing soft tissue infections.
